# Supplementary material for: A Leak-Free Inducible CRISPRi/a System for Gene Functional Studies in Plasmodium falciparum
Source: Microbiol Spectr. 2022 May 5;10(3):e02782-21. doi: 10.1128/spectrum.02782-21 (PMC9241666; doi:10.1128/spectrum.02782-21)
Supplement: SUPPLEMENTAL FILE 1 — Supplemental material. Download spectrum.02782-21-s001.pdf, PDF file, 0.6 MB [file spectrum.02782-21-s001.pdf]

Figure S1

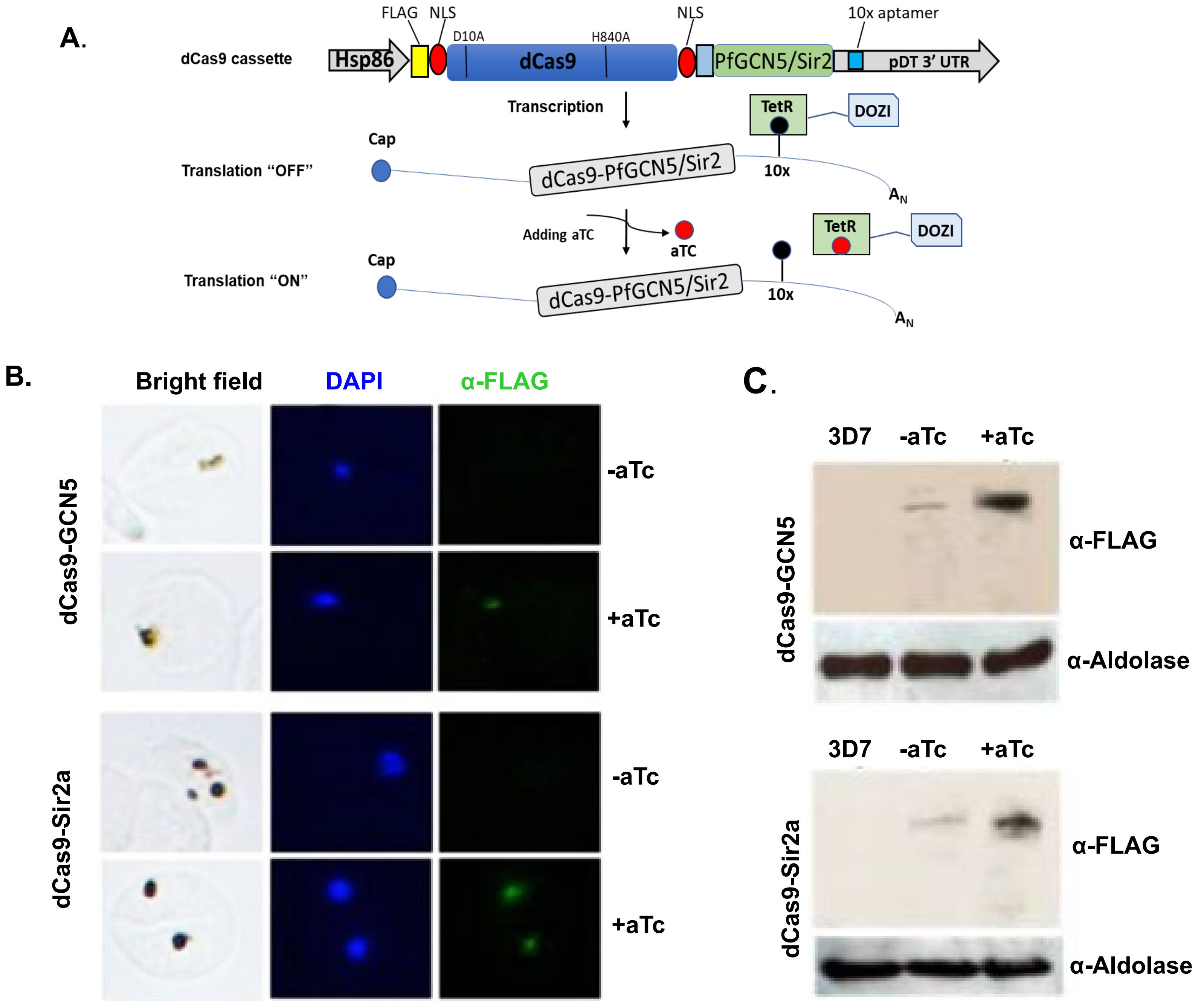

Figure S1. TetR-DOZI mediated the inducible expression of the dCas9-GCN5/Sir2a.
A. A diagram illustrates the TetR-DOZI inducible CRISPR/dCas9 system, in which 10x aptamer motifs are inserted into 3' UTR of the expression cassette. After transcription, TetR-DOZI binds aptamer motifs to block the translational process (translation "OFF"), whereas adding the tetracycline analog aTc causes TetR-DOZI to be released from the aptamer, inducing protein translation (translation "ON"). A_N indicates the polyA tail of the mRNA. **B** and **C**. immunofluorescence images (**B**) and Western blot (**C**) show efficient induction of dCas9-GCN5 (upper panel) and dCas9-Sir2a (lower panel) protein expression by the addition of aTc.

Figure S2

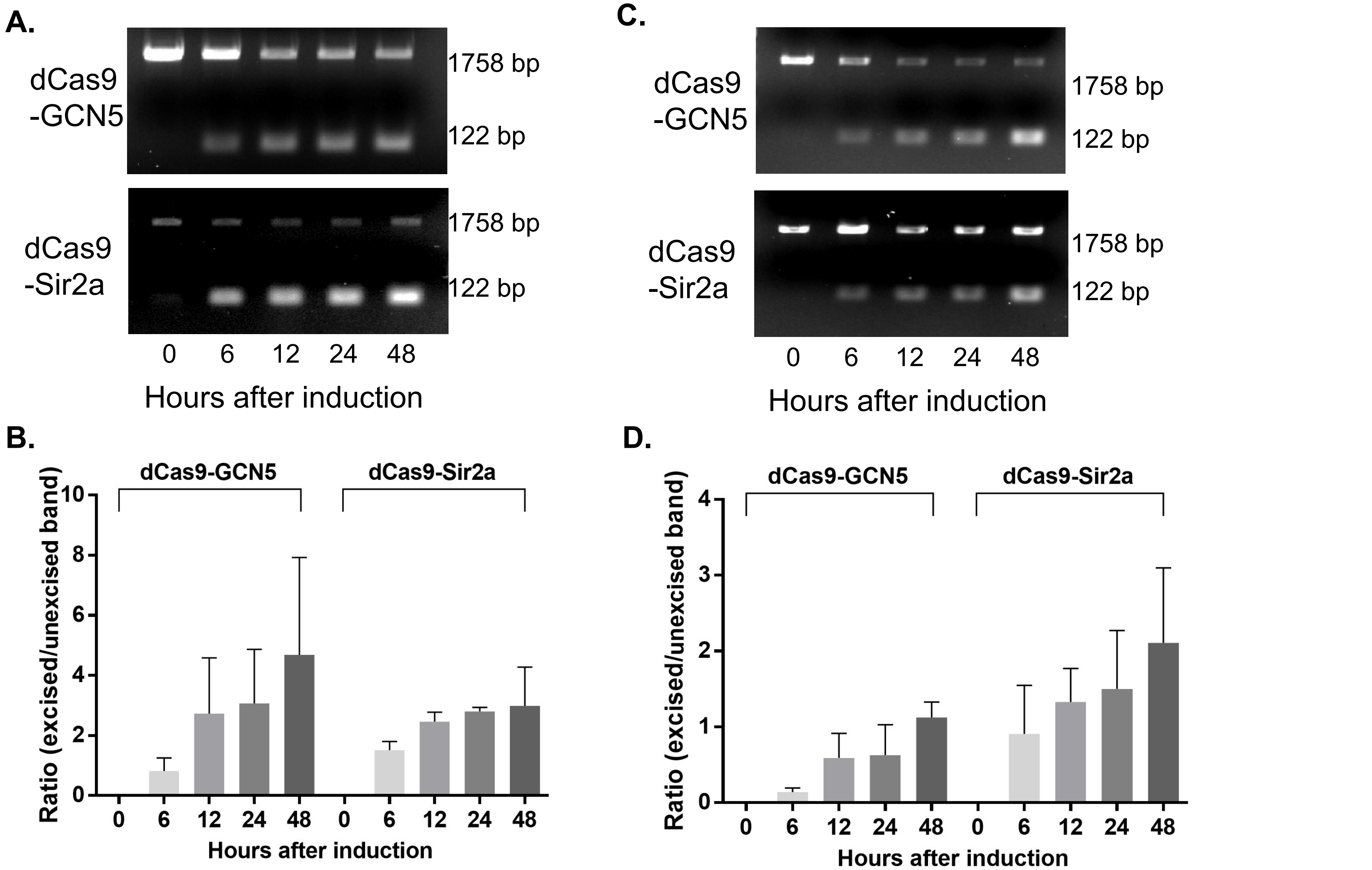

Figure S2. The dynamics of DiCre excision at trophozoite and schizont stages.
After Rap treatment at trophozoite (**A**, **B**) and schizont (**C**, **D**) stage, the efficiency of DiCre excision of *loxP*ed GFP cassette was measured by PCR with specific primers shown in Figure 1A. The gradual decrease and increase of unexcised- and excised-bands at the size of 1758bp and 122bp were shown in agarose gels (**A**, **C**), respectively. Three biological replicates were performed for the experiments in **A** and **C** to depict a bar graph showing the ratio of excised- to unexcised-bands over time elapsed after Rap treatment (**B**, **D**). The pixel intensities of DNA bands were measured using ImageJ.

Figure S3

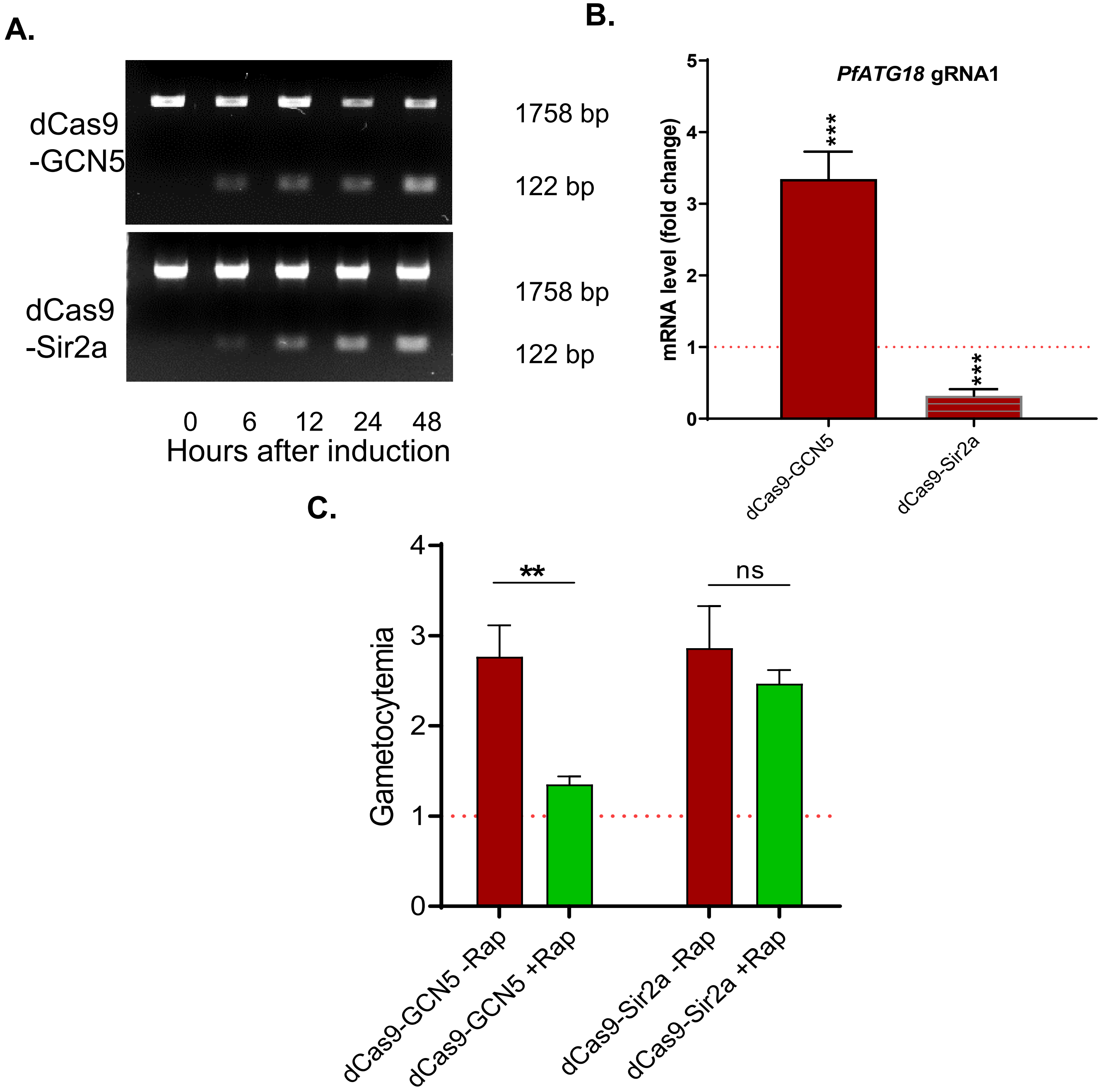

Figure S3. Regulation of *PfATG18* by the inducible dCas9 system at the sexual stage
A. The efficiency of DiCre excision of *loxP*ed GFP cassette in Day 5 (stage III) gametocytes was measured by PCR with specific primers shown in Figure 1A. The gradual decrease and increase of unexcised- and excised-bands at the size of 1758bp and 122bp were shown in agarose gels, respectively. **B**. Compared to gametocytes without Rap induction, the changes of *PfATG18* transcriptions were analyzed by RT-qPCR in dCas9-Sir2a and dCas9-GCN5 parasite lines with *PfATG18* gRNA1 48h after Rap induction. The paired T-tests were conducted (***: $P< 0.001$) from three replicates. **C**. Three replicates of gametocyte growth rates were measured after up-or down-regulation of *PfATG18* at the gametocyte stage by Rap induction (+Rap) compared to the DSMO treated (-Rap) gametocytes. (ns: not significant; **: $P< 0.01$, one-way ANOVA)**.**

Table S1 Transcriptional expression levels of eight selected genes during IDC based on the transcriptomic profile by RNAseq

The data were extracted from 3D7 iRBC RNA-seq showing the scaled TPM (transcripts per million) level (Bártfai R et al. H2A.Z demarcates intergenic regions of the plasmodium falciparum epigenome that are dynamically marked by H3K9ac and H3K4me3. PLoS Pathog. 2010 Dec 16;6(12):e1001223. doi: 10.1371/journal.ppat.1001223. PMID: 21187892)

Gene ID	Product Description	Gene Name	Hour 5	Hour 10	Hour 15	Hour 20	Hour 25	Hour 30	Hour 35	Hour 40
PF3D7_0709000	chloroquine resistance transporter	CRT	11.2	14.26	17.82	38.05	135.28	138.67	83.89	17.84
PF3D7_0727300	DNA (cytosine-5)-methyltransferase	DNMT	1.01	3.49	5.75	16.06	19.44	33.83	22.25	13.81
PF3D7_0823300	histone acetyltransferase GCN5	GCN5	8.74	12.2	16.35	46.15	220.1	228.1	276.25	108.77
PF3D7_1012900	autophagy-related protein 18	ATG18	15.27	9.86	11.29	29.47	110.81	134.58	108.69	42.64
PF3D7_1118600	histone acetyltransferase MYST	MYST	18.18	7.31	6.1	15.67	53	65	60	24.21
PF3D7_1219100	clathrin heavy chain, putative	CHC	2.18	3.58	7.45	19.08	72.64	93.72	119.21	52.05
PF3D7_1222600	AP2 domain transcription factor AP2-G	AP2-G	5.06	3.48	2.97	5.46	2.06	1.47	6.74	5.06
PF3D7_1343700	kelch protein K13	Kelch13	23.88	18.34	18.3	34.56	87.2	79.33	66.91	51.34

Table S2. TSS and gRNA locations of eight selected genes

Gene Name	Gene ID	Essential gene (Y/N)	major TSS site (-ATG bp)	sgRNA	gRNA position (-ATG bp)
MYST	PF3D7_1118600	Y	203	gRNA1	630
				gRNA2	1884
DNMT	PF3D7_0727300	N	402	gRNA1	581
				gRNA2	1696
CHC	PF3D7_1219100	Y	431	gRNA1	1420
				gRNA2	936
GCN5	PF3D7_0823300	Y	342	gRNA1	794
				gRNA2	1459
k13	PF3D7_1343700	Y	880	gRNA1	1294
				gRNA2	1600
ATG18	PF3D7_1012900	Y	1599	gRNA1	88
				gRNA2	757
				gRNA3	1409
AP2-G	PF3D7_1222600	N	489	gRNA1	176
				gRNA2	1815
				gRNA3	2779
CRT	PF3D7_0709000	Y	837	gRNA1	93
				gRNA2	509
				gRNA3	1204

Table S3. Primer information

Primer name	Sequence
**primers for plasmid construction**	
Loxp-GFP-Loxp-F	TTCGAATAAACTCGAGATAACTTCGTATAGCATACATTATACGAAGTTATGTCGACATGAGTAAAGGAGAAGAACTTTTC
Loxp-GFP-Loxp-R	TATAGTCCATCTCGAGATAACTTCGTATAATGTATGCTATACGAAGTTATCCCGGGTTATTTGTATAGTTCATCCATGC
Aptamer F	GCTGCCATATCCTTAATTAACGAGTTGTAAACTAGAGGCCTGG
Aptamer R	TACATTTTATACTTAATTAAAGGTTAACTGGCCAAGATCTCCC
Hsp86-F	ACGAAGTTATCCCGGGTTATATAATATATTTATGTACTCACAATGG
Hsp86-R	ATACAAATAACCCGGGGGCATGCTATTTGATGAATTAAC
**primers for excision event**	
Ex-F	CTTCCCACATTTCGAATAAActcg
Ex-R	GACCTTCCGCTTCTTCTTTGG
**sgRNA primers**	
AP2G sgRNA1-F	TAAGTATATAATATTGTAACTATCTATATATAAGTGTTTTAGAGCTAGAA
AP2G sgRNA1-R	TTCTAGCTCTAAAACACTTATATATAGATAGTTACAATATTATATACTTA
AP2G sgRNA2-F	TAAGTATATAATATTACATTAATAAATTCATAATAGTTTTAGAGCTAGAA
AP2G sgRNA2-R	TTCTAGCTCTAAAACTATTATGAATTTATTAATGTAATATTATATACTTA
AP2G sgRNA3-F	TAAGTATATAATATTGTGTACAACTAATAATGCACGTTTTAGAGCTAGAA
AP2G sgRNA3-R	TTCTAGCTCTAAAACGTGCATTATTAGTTGTACACAATATTATATACTTA
CRT sgRNA1-F	TAAGTATATAATATTAGTCTAAAAATATAATATATGTTTTAGAGCTAGAA
CRT sgRNA1-R	TTCTAGCTCTAAAACATATATTATATTTTTAGACTAATATTATATACTTA
CRT sgRNA2-F	TAAGTATATAATATTAACTTGGGAAAGTAAAAAAAGTTTTAGAGCTAGAA
CRT sgRNA2-R	TTCTAGCTCTAAAACTTTTTTTACTTTCCCAAGTTAATATTATATACTTA
CRT sgRNA3-F	TAAGTATATAATATTAAATAAATAATCGGAAAAATGTTTTAGAGCTAGAA
CRT sgRNA3-R	TTCTAGCTCTAAAACATTTTTCCGATTATTTATTTAATATTATATACTTA
ATG18 sgRNA1-F	TAAGTATATAATATTTGGTAAAATATATAAAAAGTTTTAGAGCTAGAA
ATG18 sgRNA1-R	TTCTAGCTCTAAAACTTTTTATATATTTTACCACAAATATTATATACTTA
ATG18 sgRNA2-F	TAAGTATATAATATTTAATAACATATAAATCAACAGTTTTAGAGCTAGAA
ATG18 sgRNA2-R	TTCTAGCTCTAAAACTGTTGATTTATATGTTATTAAATATTATATACTTA
ATG18 sgRNA3-F	TAAGTATATAATATTTAATAAGGTTTTGAAGGGTAGTTTTAGAGCTAGAA
ATG18 sgRNA3-R	TTCTAGCTCTAAAACTACCCTTCAAAACCTTATTAAATATTATATACTTA
MYST sgRNA1-F	TAAGTATATAATATTGAAAAACAATTATATGTTTAGTTTTAGAGCTAGAA
MYST sgRNA1-R	TTCTAGCTCTAAAACTAAACATATAATTGTTTTTCAATATTATATACTTA
MYST sgRNA2-F	TAAGTATATAATATTGACGCTAATGTTGAATGAACGTTTTAGAGCTAGAA
MYST sgRNA2-R	TTCTAGCTCTAAAACGTTCATTCAACATTAGCGTCAATATTATATACTTA
DNMT sgRNA1-F	TAAGTATATAATATTTATATGTGTTCAAAGGCATAGTTTTAGAGCTAGAA
DNMT sgRNA1-R	TTCTAGCTCTAAAACTATGCCTTTGAACACATATAAATATTATATACTTA
DNMT sgRNA2-F	TAAGTATATAATATTGGTTCAGTATACCATAAACAGTTTTAGAGCTAGAA
DNMT sgRNA2-R	TTCTAGCTCTAAAACTGTTTATGGTATACTGAACCAATATTATATACTTA
GCN5 sgRNA1-F	TAAGTATATAATATTATACTTAATATTATCAAATGGTTTTAGAGCTAGAA
GCN5 sgRNA1-R	TTCTAGCTCTAAAACCATTTGATAATATTAAGTATAATATTATATACTTA
GCN5 sgRNA2-F	TAAGTATATAATATTACACTATCATAATAAGTAATGTTTTAGAGCTAGAA
GCN5 sgRNA2-R	TTCTAGCTCTAAAACATTACTTATTATGATAGTGTAATATTATATACTTA
CHC sgRNA1-F	TAAGTATATAATATTATAAATAAGCAATCATTTCTGTTTTAGAGCTAGAA
CHC sgRNA1-R	TTCTAGCTCTAAAACAGAAATGATTGCTTATTTATAATATTATATACTTA
CHC sgRNA2-F	TAAGTATATAATATTAAAAAATTACCATGAAAATAGTTTTAGAGCTAGAA
CHC sgRNA2-R	TTCTAGCTCTAAAACTATTTTCATGGTAATTTTTTAATATTATATACTTA
K13 sgRNA1-F	TAAGTATATAATATTGTTATCATTCAAAAATATATGTTTTAGAGCTAGAA
K13 sgRNA1-R	TTCTAGCTCTAAAACATATATTTTTGAATGATAACAATATTATATACTTA
K13 sgRNA2-F	TAAGTATATAATATTTACCATGTTGAATTGGTGACGTTTTAGAGCTAGAA
K13 sgRNA2-R	TTCTAGCTCTAAAACGTCACCAATTCAACATGGTAAATATTATATACTTA
**primers for qPCR**	
AP2G-F	TTGTTCATGGAGATGTAGATGGTG
AP2G-R	TTTGTTCTTTGGGATTACTTTTTTT
Clathrin-F	ATACATCCGGACAATTATCTCCAC
Clathrin-R	TTGTAAAACAACCGGTTTTACCAA
DNMT-F	CAACCACTTGTTGTCATGTGGCAA
DNMT-R	TTTTGCGTTCATATGCTTGGTTGG
GCN5-F	TGTTATTACCTCTTCCTTATTGTC
GCN5-R	TTTCTTCCTCTTCATCATATTTAT
K13-F	TATTAATGTTGGTGGAGCTATTTT
K13-R	CCTTGTTTATCTCTGGTTACATGA
MYST-F	AAGAGCAAAGAAAAAACAGGTGAC
MYST-R	TAAAGCGTTTGGAAAAATTAAAGC

CRT-F	GTTGTATACAAGGTCCAGCAATAGC
CRT-R	CCAAATAGGTAGCCAAACAAAGTTACG
atg18-F	CCAGGATTATGGTTGTTTGTG
atg18-R	GTTAGTTTCGCTATTTCCCG
Gexp5-F	CCAATGGAATCATGTACACAT
Gexp5-R	GTCTATAAAATCTGTACGCGTC
Pfs16-F	CGAAAGTTCATACCATCTTTAGC
Pfs16-R	GTTGAAGGGGATCCTTTTCC
seryl-tRNA synthetase F	AAGTAGCAGGTCATCGTGGTT
seryl-tRNA synthetase R	TTCGGCACATTCTTCCATAA
